# The fate of visual working memory items after their job is done

**DOI:** 10.1167/jov.25.4.7

**Published:** 2025-04-16

**Authors:** Zachary Hamblin-Frohman, Jay Pratt

**Affiliations:** 1 Department of Psychology, University of Toronto, Toronto, Canada

**Keywords:** visual working memory, visual search, attentional templates

## Abstract

Visual working memory is a competitive, capacity-limited system for the storage of feature and object-based information. In change-detection tasks, items are encoded into memory and, after a retention period, are compared against a test set. Loss of information can occur from attentional interference or prioritizing some items over others. But what happens to the memory representations after the change-detection task is completed? The current article examines the fate of a memory item after its behavioral purpose has been fulfilled. Participants encoded a single item in memory for a difficult change-detection task. Visual search trials were presented both before and after the memory test was completed. Singleton distractors were present in these search trials that could match or not the memory item. In Experiment 1, memory-driven capture (the memory-matching distractors led to longer search response times than the unrelated distractor) was observed in the pre-memory test and, in a weaker form, the post-test search trials. In Experiment 2, we introduced cues that indicated the memory test would not occur on a subset of trials, controlling for re-exposure to the memory stimulus. Memory-driven capture was again observed for these post-cue search trials, but only at a short time interval, at a longer interval this effect was attenuated. These results suggest that the memory representations only linger briefly in the visual system.

## Introduction

Visual working memory (VWM) is a short-term storage of visual, feature, or object-based information. Often, VWM is used to retain task relevant items over short durations when those items are no longer directly perceivable. Change-detection tasks measure VWM by comparing an encoded memory item to subsequent stimuli presentations. Change-blindness performance and accuracy errors reveal that VWM capacity is limited to three or four simple items (Alvarez & Cavanagh, 2004; Cowan, 2001). Much research is dedicated to how, or which, items are encoded and maintained (e.g., Woodman & Vogel, 2008), but less is known about the fate of VWM items once they have been used and are no longer relevant. The current paper aims to examine what happens to an encoded memory item when its task relevancy is finished.

At the end of a change-detection task, a participant makes some form of response to a test array and then the next trial commences. What happens to that previously maintained information? It is possible that the information is jettisoned immediately from memory to clear space for upcoming encoding demands. Conversely, as attentional resources are removed from item retention the memory fidelity naturally fades over time. Note that we differentiate “fading” as the extinguishing of a no longer needed memory representation from previously defined temporally-based memory decay. Several VWM studies have investigated the decay of memory items that are being *actively maintained* and have noted that memory fidelity decays over the course of 10 seconds (e.g., Ricker, Spiegel, & Cowan, 2014; Zhang & Luck, 2009); however, this “resisted” decay may be a very different behavior to the memory fading of no longer relevant information. A final possibility is that the information is still stored in VWM in a fragile state (e.g., Olivers, Peters, Houtkamp, & Roelfsema, 2011), only to be overwritten with the presentation of new visual information (e.g., Woodman & Luck, 2010).

To address these three possibilities, we turn to the memory capture paradigm. Olivers, Meijer, and Theeuwes (2006) noted that a single item stored in VWM can act as a pseudo target template for visual search (see also; Olivers, 2009; Van Moorselaar, Theeuwes, & Olivers, 2014). In this study, a visual search task was presented in the retention period of a change detection task. Critically, a distractor was presented that could either match the contents of memory or was unrelated. Response times (RT) were reliably slowed by memory-matching distractors over memory-unrelated distractors. We define this effect as memory-driven capture because attention was directed towards irrelevant items that match the current contents of VWM. If there is lingering information stored in VWM after the completion of the memory task, then memory-driven capture effects should still be observed in search trials presented after the memory test is completed.

Olivers and colleagues (2006), in fact, tested this proposal and found no evidence for lingering memory driven capture when search trials were presented post-memory test. However, one aspect of their original design bears revisiting. The authors were concerned with having matching delay periods between the initial encoding of the memory item and the presentation of the search array (both pre-memory test and post-memory test search). This led to the inter-stimulus interval (ISI) between the memory test and the post-test search to be variable (dependent on the speed of the memory-test response). Participants had a total of 3000 ms to respond to the memory-test and any remaining time was added to an additional 1000 ms interval before the search display was presented. If the average memory response times were between 1000 ms and 1500 ms, this would mean there was a total ISI of 2500–3000 ms between the completion of the memory task and the presentation of the search array. This long period may have given the no-longer-relevant memory information ample time to fade or be discarded. In other words, the VWM traces from the memory may have been forgotten by the time the search display was presented.

The two experiments presented in this article aim to investigate precisely what happens to the no longer needed memory information directly after its behavioral function. Critically, we did this by using a range of ISIs that were time-locked to the completion of the memory test rather than the initial presentation. The first experiment revealed that there was a weak lingering effect of the memory-matching distractors in post-memory test search trials. Experiment 2 showed that the lingering VWM representation continued to guide behavior even after accounting for any memory-refreshing that could have occurred in the memory test phase but at a limited time-frame. Together these two experiments indicate that memory representations linger for a brief period before being removed from the visual system.

## Experiment 1

Experiment 1 investigated memory driven capture effects at variable ISIs before and after the memory test was completed. A similar paradigm to that of Olivers et al. (2006) was used; participants encoded a memory stimulus for a memory task requiring high precision. Both before and after the memory test a simple visual search was presented which could contain a distractor that either matched or was unrelated to the memory contents. Memory-driven capture can be observed via the additional distraction attributed to the memory-matching distractor compared to the unrelated distractor. There are several predictions that can be drawn for memory-driven capture effects for the post-test search from the different proposed removal strategies. First, if memory items are discarded immediately after their use has been fulfilled, then we would expect no differences between matching and unrelated distractors in the post-memory test search trials. Second, if memory items faded over time, then memory-driven capture effects should be present at the early ISIs and attenuated at the longer ISI conditions. Finally, if the memory items remain in a fragile state, then we would expect consistent memory driven capture across all ISI conditions. Critically, any memory-driven capture effects observed in the post-test search trials can be compared to the pre-test trials where the VWM item was being actively retained to assess their relative strengths.

### Methods

#### Participants

Data was collected online, hosted by Pavlovia using Psychopy language (Peirce, 2007). Participants were recruited through Prolific (www.prolific.com), all reported normal or corrected-to-normal vision and no abnormalities in colour vision. Sample size was based on the load-1 memory-driven capture effect from Van Moorselaar and colleagues (2014), *t*(14) = 3.37. To achieve a power of 85% (with 70% assurance) the BUCSS tool (Anderson, Kelley, & Maxwell, 2017) suggested a target sample size of 34 participants. Forty participants were initially recruited, four were excluded for having low search accuracy (< 55%), three participants were excluded for low memory accuracy (< 40%). This left 33 participants (13 female, 3 non-binary, *M*_age_ = 33.8) for the final analyses.

#### Stimuli

All stimuli were presented against a grey backdrop. In the interval screens a black fixation cross was presented (height: 25 pixels). For memory encoding a single circle (radius: 30 pixels) was presented at the center of the screen. There were eight categories of memory colors: red, orange, gold, green, teal, blue, purple, and pink. For each category there were four variations of color that varied by 10° in an RGB color space. The memory color was randomly selected from within each range of colors. For the memory test display three circles were presented (150 pixels to the left and right of fixation), one of which was the correct memory color, whereas the other two were randomly selected from the same category set.

**Figure 1.**
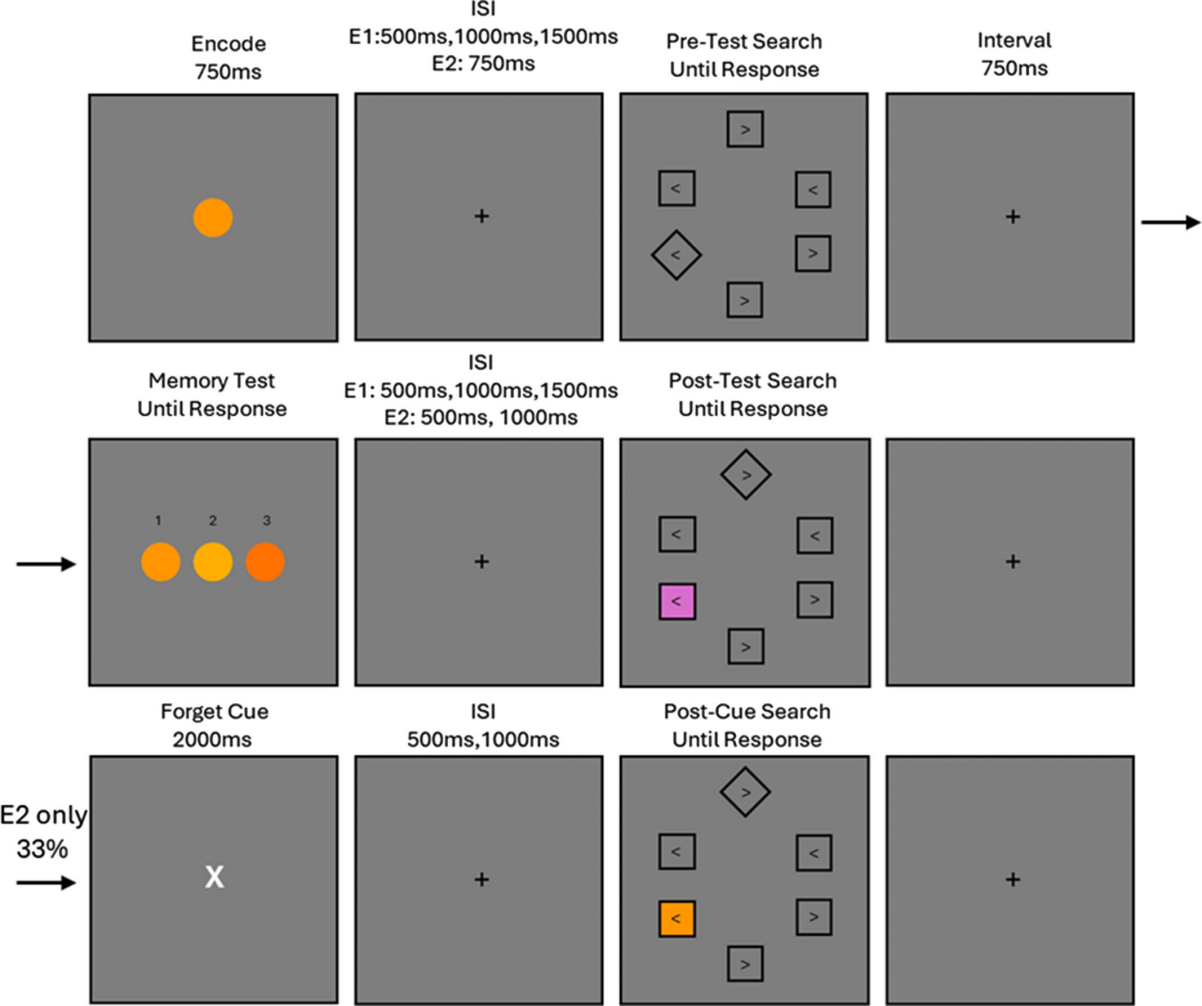
Experimental design for Experiments 1 and 2. Participants encoded a specific color hue. Before and after the memory test a visual search was performed for a diamond shaped target. A distractor was sometimes presented in either the pre- or post-memory search. The distractor could either match the memory item or was an unrelated color. In Experiment 2, on one third of trials the memory test did not occur, as indicated by a cue, but the subsequent search still happened.

In the search arrays six squares were presented (65 × 65 pixels). Two were presented on the midline 320 pixels above and below fixation. The other four were presented in a square shape ± 265 pixels to the left and right of fixation and ± 135 pixels above and below. The search target was rotated by 45°. In all shapes there was a response character “<” or “>” (height: 20 pixels). All search items except for the distractor were unfilled with a black border. The distractor could either exactly match the color of the memory item or in the unrelated condition was selected randomly from within one of the other color categories. For a visual depiction of stimuli and design see Figure 1.

#### Procedure

To begin each trial, the memory encoding display was presented for 750 ms. Next was a variable ISI of 500 ms, 1000 ms, or 1500 ms before the pre-memory search display was presented. The first search array was displayed for 2500 ms or until a response was recorded. Participants were instructed to respond to the orientation of the arrow contained within the diamond shape with the left or right arrow keys. If the response window timed out or an incorrect response was returned, error feedback was given to the participant. After the response was recorded there was another 750 ms interval until the memory test display was presented. For the memory test three items were presented, one of which was identical to the encoded item. Participants reported which item they believed to be correct using the number keys 1 through 3. After a memory response was recorded the second variable ISI phase commenced, again lasting for 500 ms, 1000 ms, or 1500 ms, before the post-test search was presented. This like the pre-test search was displayed for 2500 ms or until a response was recorded. Feedback was also given for erroneous and missing responses. After search completion there was another 750 ms interval before the next trial commenced. Feedback was not given to the participants for their memory responses outside of the practice trials.

#### Design

Participants completed a total of 440 experimental trials. Distractors could only ever be presented in one of the two search displays (pre or post memory test), never in both within the same trial. On one third of trials no distractor was present in either search display. In one third of trials a distractor appeared in the pre-memory test search (50% memory matching distractor, 50% unrelated) and in the other third the distractor appeared in the post-test search. All pre-search ISIs were equally likely and the ISIs between the first and second search were not contingent upon each other. Participants completed 10 practice trials before commencing the experiment.

### Results

For all follow-up comparisons, Bayesian tests with a default Cauchy prior of 0.707, (Morey, Romeijn, & Rouder, 2016) were used to assess the likelihood of effects and BF_10_ statistics were reported. A BF_10_ larger than 3.0 indicates moderate evidence for the alternate hypothesis over the null, whereas a BF_10_ smaller than 0.3 indicated moderate evidence for the null hypothesis (Quintana & Williams, 2018). Bayes factors are used in addition to standard difference testing to provide more information on the probabilities of effects that are reported as significant and the likelihood of true null effects.

**Figure 2.**
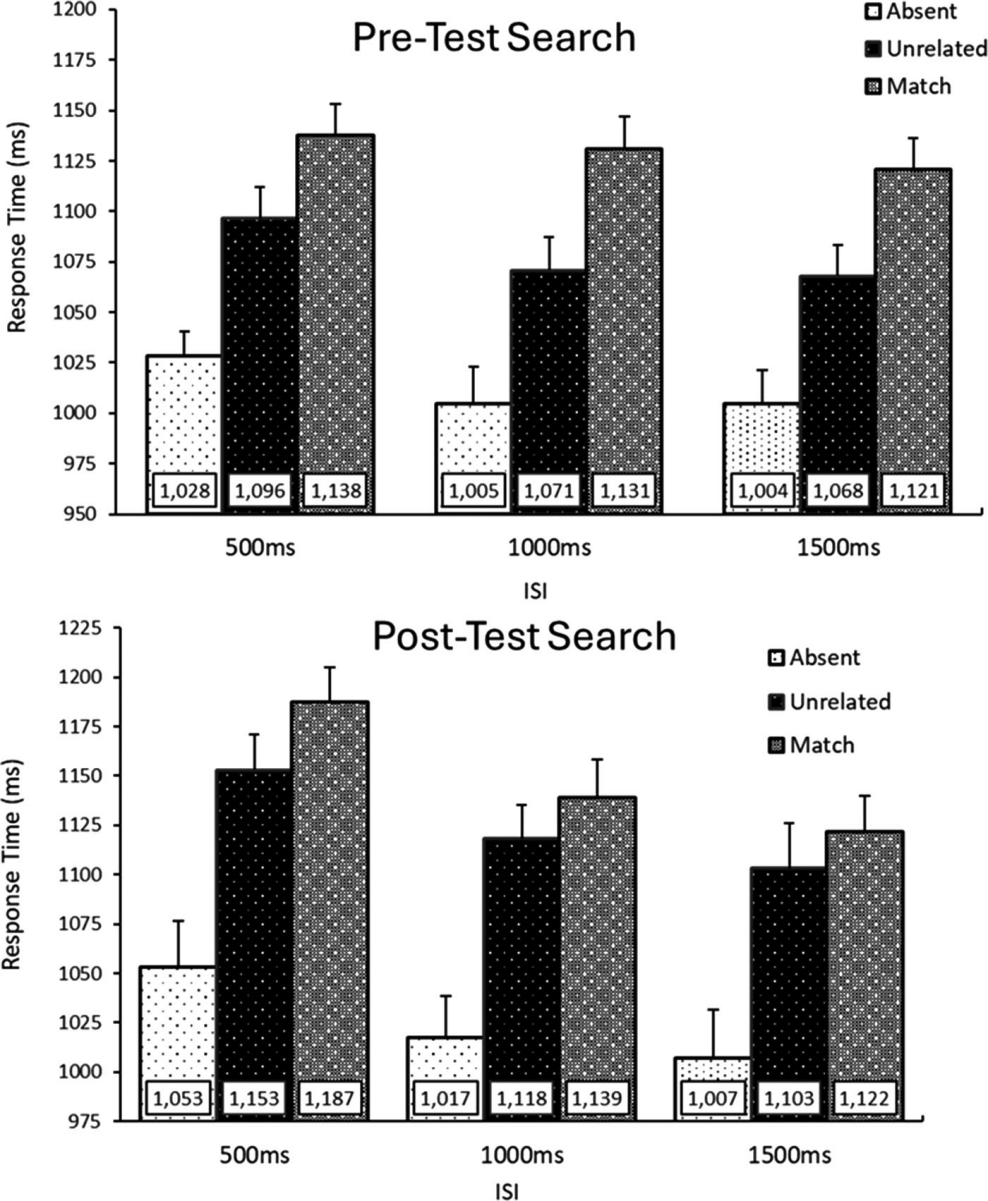
Response time (RT) results from Experiment 1. *Above*: RTs from the pre-memory test search trials. These results replicated the memory-capture effect. RTs were slower for memory-matching distractors compared to unrelated distractors. This effect was consistent across the encoding-search ISI. *Below*: Results from the post-test search trials. Overall, matching distractors led to longer RTs than unrelated distractors, however the magnitude of this effect was less than in the pre-test search trials. Error bars represent within subjects confidence intervals (Loftus & Masson, 1994).

Search accuracy was high for both pre (*M* = 95.7%) and post-test (*M* = 94.1%) search trials, and error trials were excluded from further analysis. RTs that were unusually fast or slow for each individual participant (individual mean ± 3 standard deviations) were excluded (1.3% of trials). Memory performance (*M* = 54.79%) was above chance level (33.3%), *p* < 0.001.^1^ For post-memory search an additional 1.5% of trials were excluded for abnormally short or long responses to the memory test (<500 ms or >4000 ms). All condition means are shown in Figure 2.

#### Pre-test search trials

A 3 (Distractor Type: Absent, Unrelated, Matching) × 3 (ISI: 500 ms, 1000 ms, 1500 ms) repeated measures ANOVA was run on RTs for the pre-memory test search trials. A main effect of Distractor type emerged *F*(2, 64) = 84.15, *p* < 0.001, η^2^_p_ = 0.72. Planned contrasts revealed there were longer RTs for matching distractors over unrelated distractors: *t*(32) = 6.82, *p* < 0.001, BF_10_ = 9.43 × 10^5^, and longer RTs for unrelated distractors compared to distractor absent trials: *t*(32) = 7.53, *p* < 0.001, BF_10_ = 1.45 × 10^5^. A main effect of ISI also emerged, *F*(2, 64) = 7.90, *p* < 0.001, η^2^_p_ = 0.20. This reflected a RT delay for the 500 ms ISI compared to the 1000 ms ISI, *t*(32) = 3.63, *p* < 0.001, BF_10_ = 32.54. The 1000 ms and 1500 ms ISI did not differ: *t*(32) = 0.72, *p* = 0.480, BF_10_ = 0.24. The interaction between ISI and distractor type failed to emerge as significant, *F*(4, 128) = 0.74, *p* = 0.566, η^2^_p_ = 0.02, revealing that memory-driven capture effects were consistent across ISI condition.

#### Post-test search trials

For the critical Post-test search trials, a 3 (Distractor Type: Absent, Unrelated, Matching) × 3 (ISI: 500 ms, 1000 ms, 1500 ms) repeated measures ANOVA was run on RTs for the Post-memory test search trials. A main effect of Distractor type emerged *F*(2, 64) = 72.98, *p* < 0.001, η^2^_p_ = 0.70. Planned paired t-tests revealed there were longer RTs for matching distractors over unrelated distractors: *t*(32) = 2.96, *p* = 0.006, BF_10_ = 7.02, and longer RTs for unrelated distractors compared to distractor absent trials: *t*(32) = 8.85, *p* < 0.001, BF_10_ = 2.66 × 10^6^. A main effect of ISI also emerged, *F*(2, 64) = 14.90, *p* < 0.001, η^2^_p_ = 0.32. This reflected a RT delay for the 500 ms ISI compared to the 1000 ms ISI, *t*(32) = 3.66, *p* < 0.001, BF_10_ = 35.39 and anecdotal evidence that the 1000 ms was slower than 1500 ms ISI condition: *t*(32) = 2.13, *p* = 0.041, BF_10_ = 1.36. The interaction between ISI and distractor type failed to emerge as significant. *F*(4, 128) = 0.50, *p* = 0.740, η^2^_p_ = 0.02.

#### Memory-driven distraction pre-test vs post-test search

To compare memory capture between search displays a 2 (Search Condition: Pre, Post) × 3 (ISI: 500 ms, 1000 ms, 1500 ms) repeated measures ANOVA was conducted on the additional delay from the memory-matching distractor (memory matching RTs – unrelated distractor RTs). Results revealed a main effect of search condition, *F*(1,32) = 7.58, *p* = 0.010, η^2^_p_ = 0.19, such that there were larger memory-attributed delays for the pre-test search condition (*M* = 51.5 ms) than for the post-test search (*M* = 24.5 ms); that is, that memory driven capture effects were stronger (∼27 ms) while memory items were being actively retained than after the memory test had been resolved. No effect of ISI emerged, *F*(2,64) = 0.11, *p* = 0.896, η^2^_p_ < 0.01, and the interaction failed to emerge as significant, *F*(2,64) = 1.18, *p* = 0.313, η^2^_p_ = 0.04.

#### Post-test distraction as a function of time since encoding

It is plausible that the post-test memory distraction effects were dependent on the time elapsed since the initial encoding of the memory stimulus. To investigate whether memory-driven capture (Matching Distractor RTs – Unrelated Distractor RTs) in the post-test search trials was dependent on the amount of time since the initial encoding, the trials were split into three conditions according to the post-encoding ISI and compared using paired *t*-tests. No differences were observed for any pairings, for example, memory-driven capture effects for the 1000 ms post-test ISI did not differ at any of the post-encoding ISI conditions: all *t*s(32) < 0.60, *p*s > 0.556, BF_10_s < 0.22. These results indicate that the memory-driven capture effects in the post-test search trials were not driven by variations in time since the initial exposure to the memory stimulus.

### Discussion

Experiment 1 compared memory-driven capture effects (e.g., Olivers et al., 2006) in pre-memory test search trials and post-test search trials. For the pretest search the results were as predicted; longer RTs were observed for memory-matching distractors compared to unrelated distractors at all ISI conditions. For the critical post-test search, however, a consistent distraction effect from the memory-matching distractors was observed above and beyond that of the unrelated distractors across ISI conditions. These results are in line with the fragile state hypothesis; VWM contents were not removed after task completion but were retained in a passive state and continued to bias attention.

The memory-driven capture effects were, however, weaker than that of the pretest search condition. The reduction in strength of memory capture may be due to the fact that the post-test item was no longer actively maintained in VWM but instead stored in a fragile state. Olivers et al. (2011) claimed that only actively maintained VWM items can guide attention. However, more recently it has been suggested that VWM representation influence external attention dependent on the fidelity of the stored representation (Williams, Brady, & Störmer, 2022). Thus the reduction in memory-driven capture in the post-test search may be due to the removal of active attentional resources towards memory maintenance, leading to guidance from a passive fragile VWM representation (Vandenbroucke Sligte, & Lamme, 2011).

Another possibility for the weaker memory capture effects is that the memory test itself may have caused the matching distractor capture effect in the post-search trials. Participants were required to select a memory colour out of other items. Selective attention and VWM have a bi-direction relationship (e.g., Kiyonaga & Egner, 2013) and selectively attended items are often seen as being encoded into VWM (e.g., Hamblin-Frohman, 2022; Hamblin-Frohman & Becker, 2023). Thus the selected memory test item may have been encoded into VWM, leading to additional distraction in the post-search trials.

## Experiment 2

Experiment 2 tested whether lingering memory representations can guide attention after its contents had been cued to be irrelevant. The same paradigm as Experiment 1 was used but with the addition of a small number of trials where the memory test was not performed. In these trials participants were given a cue with ample time to indicate that the memory-test would not occur on this trial and thus that the stored VWM information was no longer relevant or needed.

If the post-test memory capture was due to the re-encoding of new information from the test display, then no memory capture should be observed in the post-forget cue search trials (because there would be no memory refreshing). Conversely, if VWM representations linger in a passive state then similar memory driven capture effects should be observed in the post-cue to the post-test search trials.

### Methods

#### Participants

Data was collected online, hosted by Pavlovia using Psychopy language (Peirce, 2007). Participants were recruited through Prolific (www.prolific.com), all reported normal or corrected-to-normal vision. Thirty-nine participants were recruited, two were excluded for low search accuracy (<55%) and one for low memory performance (<35%), leaving 36 for the final analysis (16 female, two non-binary, *M* age = 31.8).

#### Stimuli and procedure

The stimuli used were identical to those in Experiment 1 (see Figure 1). The only addition was the “forget” cue that indicated the memory test was not occurring for that trial. The forget cue replaced the fixation cross with a larger (height) white X. ISIs were altered in Experiment 2. There was now only a single ISI (750 ms) before the Pre-test search, and the variable post-test (and post-cue) ISI was either 500 ms or 1000 ms. The duration of the Forget Cue was generously based on the average memory response time in Experiment 1 (*M* = 1421 ms), the cue was displayed for 2000 ms.

#### Design

There were 450 experimental trials. Distractors appeared with the same proportions as in Experiment 1, distractors were present on two thirds of all trials, evenly distributed between the first and second search displays. Fifty percent of distractors were memory matching, and 50% were unrelated. On one third of trials the memory test did not occur, instead replaced by the forgetting cue. Participants completed 10 practice trials before commencing the experiment.

### Results

Search accuracy was high for both post-test (*M* = 95.2%) and post-cue (*M* = 96.2%) search trials, error trials were excluded from further analysis. Unusually fast or slow RTs were excluded (1.8%). Memory accuracy (*M* = 54.2%) was higher than chance performance, *p* < 0.001. 1.9% of post-test trials were excluded for abnormally short or long memory RTs (<500 ms or >4000 ms).

#### Memory Capture

A one-way (Distractor type: Absent, Unrelated, Match) repeated measures ANOVA was conducted on RTs for the pre-memory test search displays. A significant effect emerged, *F*(2, 70) = 62.78, *p* < 0.001, η^2^_p_ = 0.64. Planned paired two-tailed t-tests confirmed memory capture. RTs were longer for the matching (*M* = 1043.3ms) compared to the unrelated distractors (*M* = 995.6ms), *t*(35) = 4.74, *p* < 0.001, BF_10_ = 643.77. In turn, RTs were slower for the unrelated than distractor absent trials (*M* = 934.3ms), *t*(32) = 7.83, *p* < 0.001, BF_10_ = 3.67 * 10^6^.

#### Post-test

**Figure 3.**
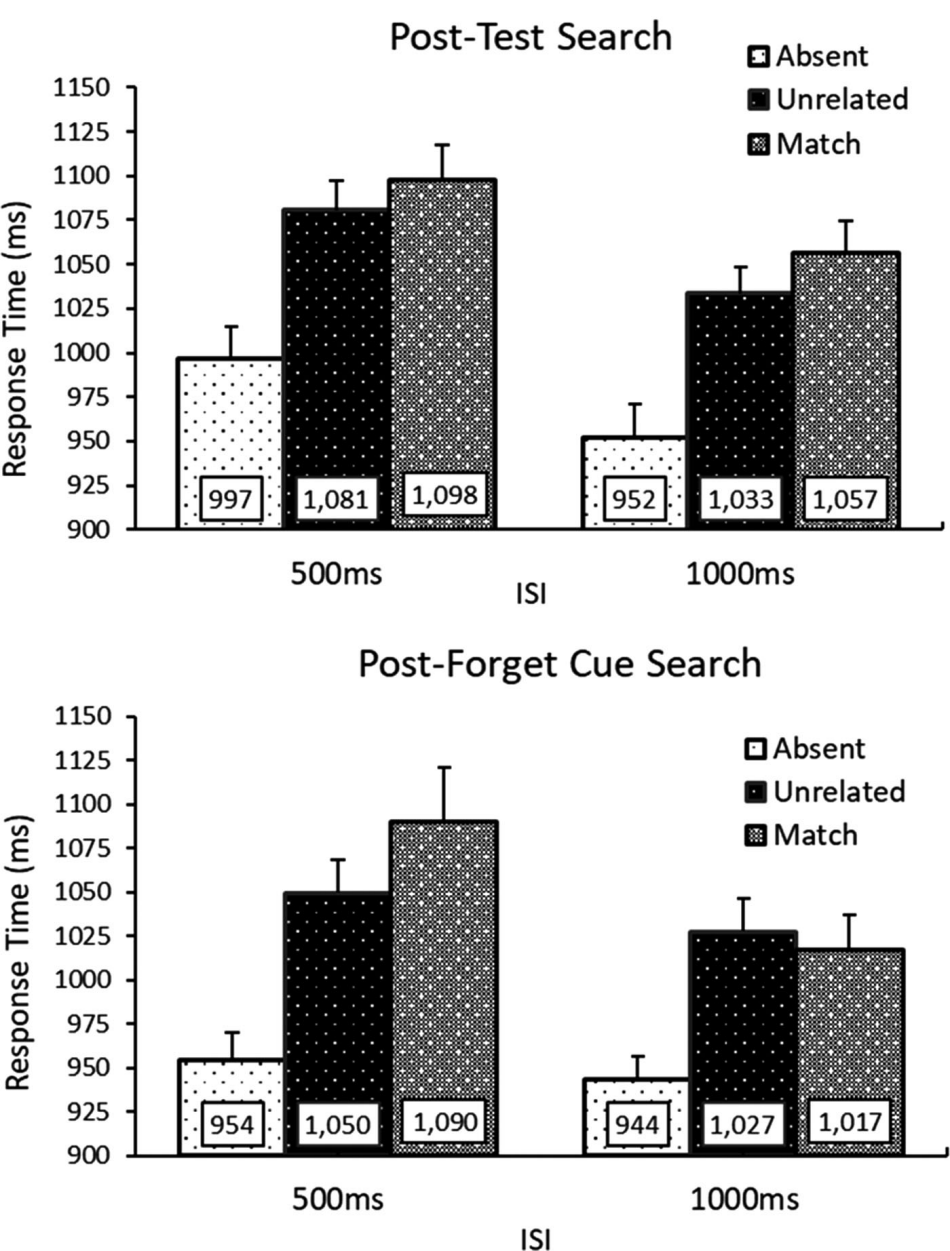
Response time (RT) results from Experiment 2. *Above*: RTs from the post-memory test search trials, replicating the effects from Experiment 1. RTs were slower for memory-matching distractors than unrelated distractors. *Below*: Results from the post-forget cue search trials. At the shorter ISI the matching distractors led to longer RTs than unrelated distractors. At the longer ISI this effect was attenuated. Error bars represent within subjects confidence intervals (Loftus & Masson, 1994).

A 3 (Distractor Type: Absent, Unrelated, Matching) × 2 (ISI: 500 ms, 1000 ms) repeated measures ANOVA was run on RTs for the Post-memory test search trials. A main effect of Distractor type emerged *F*(2, 70) = 57.78, *p* < 0.001, η^2^_p_ = 0.62. To investigate this main effect, these results were collapsed over ISI. Planned paired two-tailed *t*-tests revealed that unrelated distractors led to slower RTs than distractor absent trials: *t*(35) = 7.38, *p* < 0.001, BF_10_ = 1.04×10^6^, and matching distractors led to slower RTs than unrelated distractor trials: *t*(35) = 2.77, *p* < 0.001, BF_10_ = 4.71; condition means can be seen in Figure 3. A main effect of ISI also emerged, *F*(2, 70) = 31.07, *p* < 0.001, η^2^_p_ = 0.47, with longer RTs for the 500 ms ISI condition. The Distractor x ISI interaction did not emerge as significant, *F*(2, 70) = 0.08, *p* = 0.922, η^2^_p_ < 0.01, reflecting that the additional search cost of the matching distractors remained consistent over ISI.

#### Post-cue

A 3 (Distractor Type: Absent, Unrelated, Matching) x 2 (ISI: 500 ms, 1000 ms) repeated measures ANOVA was run on RTs on post-Forget cue search trials. A significant main effect of Distractor type *F*(2, 70) = 53.92, *p* < 0.001, η^2^_p_ = 0.61, and ISI emerged, *F*(2, 70) = 19.75, *p* < 0.001, η^2^_p_ = 0.36. Importantly, these effects were qualified by a Distractor x ISI interaction, *F*(2, 70) = 7.30, *p* = 0.001, η^2^_p_ = 0.17. Planned paired *t*-tests revealed that at the 1000 ms interval, there was satisfactory evidence for no difference between RTs for matching and unrelated distractors, *t*(35) = 0.74, *p* = 0.467, BF_10_ = 0.23. Conversely, at the shorter 500 ms ISI there was significant, but weak, evidence that the matchings distractors led to longer RTs, *t*(35) = 2.34, *p* = 0.025, BF_10_ = 1.96.

#### Memory-driven distraction post-cue versus post-test search

To compare memory capture between search displays, a 2 (Search Condition: Post-Test, Post-Cue) × 2 (ISI: 500 ms, 1000 ms) repeated measures ANOVA was conducted on the additional delay from the memory-matching distractor (memory matching – unrelated distractor). Results revealed a main effect of search condition, *F*(1,35) = 0.06, *p* = 0.806, η^2^_p_ < 0.01. No effect of ISI emerged, *F*(1,35) = 2.96, *p* = 0.094, η^2^_p_ = 0.08. Importantly, the interaction was significant, *F*(1,35) = 5.29, *p* = 0.028, η^2^_p_ = 0.13. Follow-up paired *t*-tests were conducted on memory driven capture effects across ISI condition for each search type. Interestingly, there was reliably less memory-capture for the post-cue search trials in the 1000 ms than the 500 ms interval, *t*(35) = 2.90, *p* = 0.006, BF_10_ = 6.22, whereas there was no difference in the effects ISI for the post-test search trials, *t*(35) = 0.34, *p* = 0.733, BF_10_ = 0.19.

### Discussion

Experiment 2 tested lingering memory-driven capture under two scenarios; when the to-be-forgotten item was responded to in the memory test phase and when a cue was given to indicate the memory contents was no longer relevant. The results of the post-memory test search trials replicated those from Experiment 1; memory capture effects were observed at all ISIs. However, unlike the first experiment, memory capture was only observed for the post-forget cue trials at the 500 ms ISI. At 1000 ms memory-driven capture was attenuated for the post-cue search trials, suggesting that at this point the item had been completely removed from memory. The findings of the post-cue search trials reveal that in memory items are in fact removed from VWM over a brief period. Importantly, this also shows that the post-test memory capture from Experiment 1 may indeed have been contaminated by the memory test itself. When no stimulus refreshing or recoding was available, the memory item influenced attention at the short ISI, but over time its effect was attenuated. Thus there appears to be a quick acting forgetting process for the contents of VWM after it had been deemed irrelevant.

## General discussion

Visual Working Memory (VWM) is a capacity limited system which must be tailored efficiently to fulfill behavioral objectives. Extensive research has investigated the form of VWM representations (e.g., Woodman & Vogel, 2008) and how maintained items can influence behavior (e.g., Van Moorselaar et al., 2014) and vice versa (e.g., Hamblin-Frohman & Becker, 2023). A single item maintained in VWM can form a pseudo target template, which can guide attention to matching perceivable items (Olivers, 2009; Olivers et al., 2006). In the current study we show that this representation can continue to guide attention for a limited period after it is no longer relevant to VWM.

The current experiments measured memory driven capture effects (e.g., Olivers et al., 2006) in visual search trials when the memory-item was no longer behaviorally relevant. In the first experiment, we compared distraction from memory-matching items in search arrays presented before and after the memory test screen. Differing from Olivers and colleagues (2006; Experiment 5), we observed additional capture for the memory-matching distractors (compared to unrelated items) in the post-test search. Overall, the ISIs used in the current experiment were shorter than those from Olivers et al. (2006). A fading view of forgetting or a delayed-removal process (e.g., Tsubomi, Fukuda, Kikumoto, Mayr, & Vogel, 2024) reconciles these results. At shorter ISIs the memory representation is still present and can influence attention, but after time this representation is attenuated to a point where it can no longer influence behavior. It should be noted that this effect was dramatically weaker than the memory capture effects presented before the memory test (i.e., while it was actively maintained). Information actively retained in VWM forms an attentional template and guides attention (Desimone & Duncan, 1995; Olivers et al., 2011), and it seems clear that the distractor capture observed in the post-test search trials *did not* meet this requirement. If so, there should have been equivalent memory-driven capture between pre- and post-test search trials. Although robust memory-capture was not observed in the post-test search trials, there was still lingering distraction from the memory-related items.

An alternative explanation from lingering representations, was that the memory test display led to incidental re-encoding of the selected item back into VWM (e.g., Hamblin-Frohman & Becker, 2023) or that the test-response simply biased attention to the memory colour. The second experiment controlled for memory-item refreshing by including “forget” cues, which indicated that the memory test would not occur. These cue trials were compared against the post-test search trials used in Experiment 1. Shortly following the forget cue at the 500 ms, the memory-related distractor continued to attract attention to the same extent as the 500 ms ISI post-test search. Importantly, participants were not exposed to the memory stimulus a second time in the post-cue search, meaning that the bias toward the distractor must be attributed to the initial memory representation. Interestingly, at the longer 1000 ms ISI, the memory-matching distraction effect for the post-cue search was attenuated whereas the post-test search effect persisted. This suggests that there was in fact refreshing of the encoded memory item in the test display, whether it was re-encoding (e.g., Hamblin-Frohman & Becker, 2023) or feature priming (e.g., Kristjánsson & Campana, 2010). However, these effects cannot explain the memory-driven capture occurring in the post-cue search trials.

When the forget-cue was presented in Experiment 2, the results indicated that attentional resources could be removed from the maintenance of the memory item. The results show that the representation of the memory item degraded over time, eventually to a point where it could no longer influence behavior. Several theories posit that attentional resources are required for active maintenance of VWM items. Whether this active maintenance is sustained attention (Chun, 2011; Kiyonaga & Egner, 2013) or attentional refreshing (Barrouillet & Camos, 2021), all agree that when resources are removed or disrupted the memory items are vulnerable to forgetting. In retro-cue studies, resources are shifted away from one memory item to others leading to loss of fidelity for that item (Gunseli, Van Moorselaar, Meeter, & Olivers, 2015; Pertzov, Bays, Joseph, & Husain, 2013). Filler tasks in maintenance phases can occupy attention leading to loss of memory information (e.g., Ricker & Cowan, 2010). Importantly, the current study adds a novel perspective to how items are removed from VWM. In previous studies, attentional resources were removed from the memory item, *but always diverted to another,* be it other competing memory items or distracting filler task. The current study shows that this removal occurs for memory items that are simply no longer relevant.

There are a few things to note regarding the current study. One is that only a single memory item was encoded by participants, resulting in a very low memory load. In real-world tasks, observers display a tendency to underutilize their VWM capacity, preferring less efficient but low-VWM load strategies (Draschkow, Kallmayer, & Nobre, 2021; Somai, Schut, & Van Der Stigchel, 2020). It is plausible that when only a single item is stored in memory there is no incentive to engage in an active removal process, as there is sufficient VWM resources available to interact with any subsequent visual information. Thus, there would be more incentive to remove memory items when memory load is high. Another thing to note was the high-memory precision required by the test displays. We used these to ensure that participants encoded the stimuli in a visual, not verbal, manner (Olivers et al., 2006). However, recent studies have placed an emphasis on the importance of memory fidelity on representational strength of memory items. Williams and colleagues (2022) argue that memory items are stored in a spectrum depending on their representational fidelity, with higher memory loads leading to less memory fidelity. In the current experiment a single item was retained, and task demands required extreme memory precision. These factors may have caused the memory item to be deeply entwined into VWM leading to more difficulty in removing potential memory traces. Thus inferring any forgetting strategy may be hard to dissociate completely from the specific task demands and stimuli used. Memory arrays of different set sizes and task types may in fact result in a different pattern of forgetting behavior.

One last thing to note is that a recent study by Tsubomi and colleagues (2024) assessed a similar question using electroencephalogram (EEG) recordings. The authors measured contralateral delay activity (CDA), the neural marker for visual working memory (Vogel & Machizawa, 2004). Typically, the CDA is measured during the retention period of a change detection task, however, Tsubomi and colleagues (2024) examined the CDA after the memory test had been completed. The authors found that the CDA extinguished over the course of approximately 1000 ms. In another experiment, the authors used an auditory cue to indicate the memory test would not occur for that trial. Once again, they observed a quick attenuation of the CDA. Like the current Experiment 2, this displayed the fate of memory items without any possibility of the refreshing of the memory trace for the re-presentation of the stimulus. Tsubomi and colleagues claimed that their results were evidence for a spontaneous, strategic removal of VWM contents. However, these findings may be explained by the removal of active memory maintenance (Awh & Vogel, 2020) from the memory items and not an explicit item removal mechanism. To address this, in two of their experiments Tsubomi and colleagues (2024) used surprise trials (one trial per participant) to gain a behavioral measure of the no longer relevant memory item. They found that memory performance on these trials was substantially lower than baseline. This, they argued, was evidence that converged with their EEG findings to support a spontaneous item removal mechanism. Although the surprise trials assessed participant's explicit memory for the extinct memory item, it did not measure whether there were any remaining implicit memory traces (for an example see; Harrison, Kang, & Wilson, 2021). The current study provides corroborating evidence with the findings of Tsubomi and colleagues (2024). We show that when no memory trace refreshing is available (post-cue search trials; Experiment 2) implicit guidance occurs initially, but quickly attenuates.

In our opinion this does not necessarily dictate that item removal from memory is an active strategic process. Tsubomi and colleagues (2024) compared their results to memory decay occurring while active maintenance was still required by task demands (Ricker et al., 2014; Zhang & Luck, 2009). Yet, it is entirely plausible that there is a different rate of “fading” for memory items that are not being actively maintained. In our post-cue search trials, we show that a “forgotten” memory item influences attention at 500 ms post-cue but not at 1000 ms; although this information may not be able to be explicitly reported, as in Tsubomi et al (2024), it still has a memory representation that can be observed through implicit measures. One possibility is that the presentation of our search displays interfered with the proposed removal mechanism. At the short ISI (post-cue search) the removal process may still be in process, resulting in the observed memory-driven capture, whereas at 1000 ms the memory item had been removed. To conclude, the VWM forgetting observed here corroborates with other studies investigating VWM item removal. Visual memories are swiftly removed once their behavioral function has been fulfilled (in some manner). However, before the memories completely disperse, implicit measures can capture a lingering internal representation that can influence attentional behavior. It remains an open question, and perhaps a fruitful avenue of research, whether the removal of memory items is due to a passive, rapid-fading of representation or an active discarding process.

## Footnotes

1 There were no changes in memory-driven capture effects in the post-test search trials (Matching Distractor RTs – Unrelated Distractor RTs) when comparing, using two-tailed paired *t*-tests, between memory correct and memory incorrect trials for each ISI: all *t*s(32) < 0.86, *p*s > 0.395, BF_10_s < 0.26.
